# Longitudinal Assessment of Facial Hyperhidrosis Management: Evaluating the Utility and Quality of Life Improvements following Botulinum Toxin Injection

**DOI:** 10.3390/toxins16010059

**Published:** 2024-01-21

**Authors:** Catalin Prodan-Barbulescu, Luca Castiglione, Sonia Roxana Burtic, Marius Murariu, Shruta Reddy, Ovidiu Rosca, Felix Bratosin, Camelia Melania Fizedean, Pavel Krupyshev, Ileana Enatescu

**Affiliations:** 1 Doctoral School, “Victor Babes” University of Medicine and Pharmacy Timisoara, Eftimie Murgu Square 2, 300041 Timisoara, Romania; catalin.prodan-barbulescu@umft.ro (C.P.-B.); dr.soniaburtic@umft.ro (S.R.B.); 2 IInd Surgery Clinic, “Victor Babes” University of Medicine and Pharmacy Timisoara, Eftimie Murgu Square 2, 300041 Timisoara, Romania; 3 Department I-Discipline of Anatomy and Embryology, Faculty of Medicine, “Victor Babes” University of Medicine and Pharmacy Timisoara, Eftimie Murgu Square 2, 300041 Timisoara, Romania; 4 Department of General Surgery, “Victor Babes” University of Medicine and Pharmacy Timisoara, Eftimie Murgu Square 2, 300041 Timisoara, Romania; murariu.marius@umft.ro; 5 Research Center for Medical Communication, Victor Babes University of Medicine and Pharmacy Timisoara, Eftimie Murgu Square 2, 300041 Timisoara, Romania; 6 Department of General Medicine, SVS Medical College, Yenugonda, Mahbubnagar 509001, Telangana, India; shrutareddy097@gmail.com; 7 Department of Infectious Diseases, “Victor Babes” University of Medicine and Pharmacy Timisoara, Eftimie Murgu Square 2, 300041 Timisoara, Romania; rosca.ovidiu@umft.ro (O.R.); felix.bratosin@umft.ro (F.B.); 8 Methodological and Infectious Diseases Research Center, Department of Infectious Diseases, “Victor Babes” University of Medicine and Pharmacy Timisoara, Eftimie Murgu Square 2, 300041 Timisoara, Romania; fizedean.camelia@umft.ro; 9 Faculty of General Medicine, I.M. Sechenov First Moscow State Medical University, Bolshaya Pirogovskaya Ulitsa 2, 119435 Moscow, Russia; krupyshev1002@gmail.com; 10 Department of Obstetrics and Gynecology, Discipline of Childcare and Neonatology, “Victor Babes” University of Medicine and Pharmacy Timisoara, Eftimie Murgu Square 2, 300041 Timisoara, Romania; enatescu.ileana@umft.ro

**Keywords:** hyperhidrosis, botox, quality of life, longitudinal studies

## Abstract

Facial hyperhidrosis is a debilitating condition that can severely impact the quality of life. This study aimed to assess the long-term utility of Botulinum toxin type A therapy (BTA) for facial hyperhidrosis and its impact on quality of life over a one-year period. Conducted at the Pius Brinzeu Clinical Emergency Hospital in Timisoara, Romania, this longitudinal observational study involved 77 adult patients with primary facial hyperhidrosis. Participants received two sessions of Botulinum toxin injections (50 U IncoBTX-A each) and were evaluated at baseline, 6 months, and 12 months using the Hyperhidrosis Disease Severity Scale (HDSS), WHOQOL-BREF, Dermatology Life Quality Index (DLQI), and a bespoke survey. The study demonstrated significant reductions in HDSS scores from 3.6 ± 0.5 to 1.2 ± 0.8 post-treatment, sustained at 1.3 ± 0.6 at 12 months (*p*-value < 0.001). DLQI scores markedly decreased from 24.8 ± 4.2 to 6.2 ± 2.1 post-treatment, stabilizing at 6.5 ± 2.5 at 12 months (*p*-value < 0.001). Sweat production significantly dropped from 0.75 g ± 0.15 to 0.18 g ± 0.07 per 15 min (*p*-value < 0.001). WHOQOL-BREF scores improved notably in the mental domain from 66.7 ± 6.1 to 70.8 ± 5.2 at 12 months (*p*-value < 0.001), with physical and social domains also showing significant improvements. Correlation analysis revealed strong negative correlations between DLQI total score and HDSS (rho = −0.72, *p*-value < 0.001) and sweat production (rho = −0.68, *p*-value < 0.001). Regression analysis indicated significant predictors for DLQI total score, including HDSS (B Coefficient = −3.8, *p*-value < 0.001) and sweat production (B Coefficient = −2.2, *p*-value < 0.001). BTA therapy significantly improved the quality of life in facial hyperhidrosis patients, with lasting effects on symptom severity, sweat production, and quality of life domains. The correlation and regression analyses further substantiated the treatment’s impact on both physical and psychological aspects. These findings advocate Botulinum toxin as a viable long-term treatment for facial hyperhidrosis.

## 1. Introduction

Facial hyperhidrosis, characterized by excessive sweating of the face, is a condition that affects numerous individuals globally, leading to significant social, emotional, and psychological distress [1,2]. This condition, often underdiagnosed, can profoundly impact one’s quality of life, leading to embarrassment, social withdrawal, and decreased self-esteem [3]. The etiology of facial hyperhidrosis is multifaceted, involving overactive sympathetic nerves, but can also be idiopathic or secondary to other medical conditions [4]. Its prevalence spans various demographics, making it a universal challenge in dermatology and neurology [5].

The conventional management of facial hyperhidrosis includes topical agents, oral medications, and iontophoresis, but these treatments often yield incomplete and temporary relief [6,7]. Invasive procedures, such as sympathectomy, while effective, carry risks of complications and are not universally accepted [8,9]. Therefore, there is an increasing interest in minimally invasive yet effective treatments. Botulinum toxin type A (BTA), a neurotoxin with anticholinergic properties, has emerged as a promising therapy for this condition [10].

The mechanism of action of Botulinum toxin involves the inhibition of acetylcholine release at the neuromuscular junction, leading to a temporary reduction in sweating [11]. In the context of facial hyperhidrosis, BTA injections into the affected areas can significantly reduce sweat production, improving comfort and social interactions [12]. Several studies have highlighted the utility of BTA in reducing symptoms of hyperhidrosis, with a favorable safety profile [13,14].

Despite the proven effectiveness of BTA in managing hyperhidrosis, there is a limited understanding of its long-term impact on hyperhidrosis in association with patients’ quality of life [3]. Quality of life is a multifaceted construct that encompasses physical, psychological, and social well-being [15]. In facial hyperhidrosis, persistent discomfort and social stigma can lead to long-standing psychological impacts, affecting overall life satisfaction and social functioning [16]. Hence, assessing the quality of life is essential to fully appreciate the benefits and limitations of BTA as a therapeutic option.

This research is designed to perform an extensive longitudinal study over one year to assess the quality of life among patients with facial hyperhidrosis undergoing treatment with Botulinum toxin injections. The underlying assumptions of this study are that BTA injections markedly enhance the quality of life for individuals suffering from facial hyperhidrosis and that these enhancements persist throughout the year following the treatment. The study’s goals are to observe shifts in physical symptoms, mental health, social engagement, and overall satisfaction with life before and after BTA intervention, offering a comprehensive view of its prolonged effectiveness in treating facial hyperhidrosis.

## 2. Results

Table 1 presents the background characteristics of the 77 patients participating in the study focused on assessing the management of facial hyperhidrosis with Botulinum toxin therapy. The average age of these individuals was 32.5 years, with a standard deviation of 9.8, indicating a relatively young cohort. Gender distribution showed a slight female predominance, with 54.5% (42 patients) being female and 45.5% (35 patients) male. The majority of participants resided in urban areas, accounting for 61.0% (47 patients), compared to 39.0% (30 patients) living in rural settings. When considering relationship status, a significant majority, 67.5% (52 patients), were either in a relationship or married, while 32.5% (25 patients) were single or divorced.

In terms of employment or educational status, 64.9% (50 patients) were working, 19.5% (15 patients) were currently studying, and 15.6% (12 patients) were neither working nor studying. The average duration of symptoms among participants was 18.2 months, with a standard deviation of 12.4 months, suggesting a wide range of symptom duration prior to the study. Regarding the severity of hyperhidrosis, as measured by the Hyperhidrosis Disease Severity Scale (HDSS), the cohort was almost evenly split. Slightly more than half of the patients, 51.9% (40 patients), experienced less severe symptoms (HDSS < 3), while 48.1% (37 patients) reported more severe symptoms (HDSS ≥ 3). This distribution provides a comprehensive view of the varied severity of facial hyperhidrosis among the participants.

In the longitudinal assessment of Botulinum toxin therapy for facial hyperhidrosis, Table 2 reveals statistically significant improvements in specific aspects of quality of life. The severity of facial sweating showed a substantial reduction from a baseline score of 7.5 (±1.2) to 4.2 (±0.9) post-treatment, with this improvement largely sustained over a year (*p*-value < 0.001). Similarly, sweating management improved significantly from 5.1 (±2.3) before treatment to 6.8 (±1.4) after treatment and remained improved at 12 months (*p*-value < 0.001). The impact on daily activities also showed a significant decrease from 6.4 (±1.6) pre-treatment to 4.9 (±2.1) post-treatment, indicating a lasting positive effect on daily functioning (*p*-value < 0.001). Notably, self-confidence experienced a remarkable increase from 5.9 (±1.2) before treatment to 7.7 (±1.3) after treatment, with this gain maintained at the 12-month follow-up (*p*-value < 0.001).

In the physical domain, there was a slight yet statistically significant improvement over the one-year period. The mean score initially was 64.5 before treatment, slightly increasing to 65.3 post-treatment, dipping to 64.0 at 6 months, but eventually rising to 66.5 at 12 months (*p*-value = 0.011), suggesting a modest but significant improvement in the physical aspects of quality of life for patients. The mental domain showed a more pronounced improvement. Starting at 66.7 before treatment, the score increased to 70.2 at 1 month after treatment, continued to improve to 71.5 at 6 months, and remained high at 70.8 at 12 months (*p*-value < 0.001), suggesting that the treatment had a substantial and sustained positive impact on the mental well-being of the patients.

**Figure 1:**
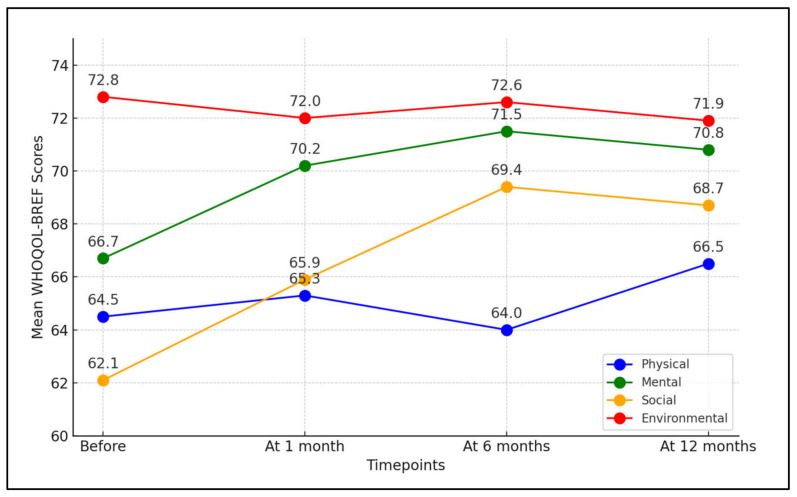
Time-based evaluation of quality of life using WHOQOL-BREF questionnaire scores.

Significant improvements were also observed in the social domain. The initial score of 62.1 improved to 65.9 at 1 month post-treatment, increased further to 69.4 at 6 months, and slightly decreased to 68.7 at 12 months, yet remained significantly higher than the baseline (*p*-value < 0.001). This demonstrates that Botulinum toxin therapy significantly enhanced the social aspects of quality of life. In contrast, the environmental domain did not show a statistically significant change throughout the study period. The scores fluctuated slightly around the baseline of 72.8, indicating that the treatment had no significant long-term impact on this aspect of quality of life (*p*-value = 0.703), as seen in Table 3 and Figure 1.

The mean score experienced a marked decline from 24.8 pre-treatment to 6.2 one month after treatment. This notable enhancement persisted, with figures showing 5.9 at 6 months and a minor rise to 6.5 at 12 months, all substantially below the initial score (*p*-value < 0.001), denoting a significant enhancement in perceived life quality post-treatment. Regarding the HDSS questionnaire scores, there was a significant reduction, indicating a marked decrease in symptom intensity. The average score fell from 3.6 (±0.5) before treatment to 1.2 (±0.8) after treatment, and this improvement was sustained at 1.1 (±0.7) at 6 months and 1.3 (±0.6) at 12 months (*p*-value < 0.001), demonstrating a substantial and continuous alleviation in hyperhidrosis symptoms following Botulinum toxin therapy.

Furthermore, sweat output, quantified in grams every 15 min, exhibited a significant decline, where the mean sweat output reduced from 0.75 g (±0.15) before treatment to 0.18 g (±0.07) post-treatment, with a further drop at 6 months (0.16 g ± 0.05) and a small increase at 12 months (0.20 g ± 0.08), still notably less than the initial measurement (*p*-value < 0.001), as indicated in Table 4. This decrease in sweat output confirms the subjective enhancements noted in DLQI and HDSS scores.

A robust inverse relationship was detected between the DLQI total score and HDSS (rho = −0.72, *p*-value < 0.001), demonstrating that a decline in hyperhidrosis severity leads to better quality of life. Additionally, a significant inverse correlation exists between the DLQI score and sweat output (rho = −0.68, *p*-value < 0.001), implying that lower sweat production enhances life quality. Conversely, HDSS positively correlates with sweat production (rho = 0.65, *p*-value < 0.001), showing that increased severity aligns with higher sweat levels.

**Figure 2:**
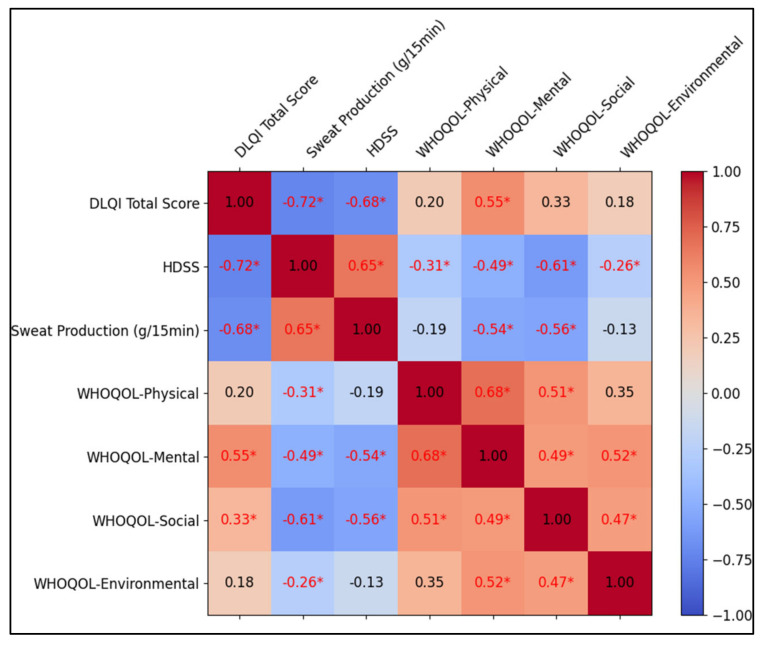
Correlation analysis matrix. Asterisk (*) represents statistical significance (*p*-value < 0.05).

In the context of WHOQOL domains, the mental domain of WHOQOL positively correlates with DLQI (rho = 0.55, *p*-value < 0.001) but inversely with HDSS (rho = −0.49, *p*-value 0.002) and sweat production (rho = −0.54, *p*-value 0.002). This indicates that an enhanced mental state is associated with less severe hyperhidrosis and reduced sweating. The social domain of WHOQOL also inversely correlates with HDSS (rho = −0.61, *p*-value < 0.001) and sweat production (rho = −0.56, *p*-value < 0.001), suggesting that social well-being improves with lower hyperhidrosis severity and sweat output, as illustrated in Table 5 and Figure 2.

The Hyperhidrosis Disease Severity Scale (HDSS) exhibited a pronounced adverse effect on life quality, marked by a B coefficient of −3.8 and a *p*-value < 0.001, underscoring that greater hyperhidrosis severity corresponds with diminished life quality. In a similar vein, increased sweat output, recorded in grams every 15 min, adversely influenced the DLQI score, evidenced by a B coefficient of −2.2 and a *p*-value < 0.001, denoting that more sweat production is linked with lower life quality.

**Figure 3:**
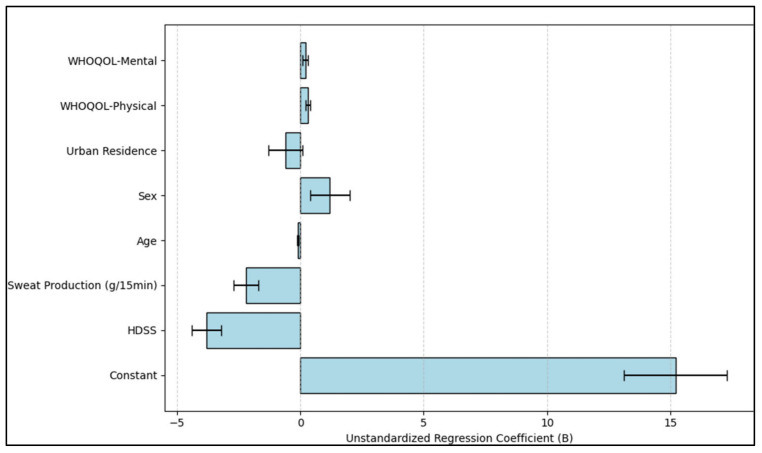
Regression analysis for DLQI total score.

Additionally, age played a crucial role, with a B coefficient of −0.1 and a *p*-value of 0.001, hinting that younger age is typically associated with a higher quality of life based on DLQI scores. The physical and mental domains of the WHOQOL survey also proved to be impactful. The physical domain, with a B coefficient of 0.3 (*p*-value 0.003), and the mental domain, with a B coefficient of 0.2 (*p*-value 0.046), both indicate that enhancements in these life quality areas relate to an improved dermatological quality of life, as illustrated in Table 6 and Figure 3.

## 3. Discussion

This study is pivotal in understanding the multifaceted impact of BTA injections in managing facial hyperhidrosis, a condition that significantly affects physical, psychological, and social well-being. Our study positions BTA injections as a viable secondary treatment option for facial hyperhidrosis, recommended after initial conservative measures such as topical antiperspirants and oral medications fail. Moreover, BTA injections were followed by no significant complications identified in our cohort of patients. BTA injections, administered in two sessions approximately six months apart, are considered for patients who exhibit persistent symptoms impacting their quality of life despite prior treatments. Moreover, the cost of treatment is dictated by the dose of BTA used, as two sessions of 50 U IncoBTX-A dose per session proved to be sufficient. The selection of this specific dose and regimen was based on a comprehensive review of the existing literature, which indicated that this dosage effectively balances efficacy and safety for treating facial hyperhidrosis [4,17,18]. Additionally, this dosing strategy was validated by our previous empiric observations, where it demonstrated a significant reduction in symptoms with minimal adverse effects, thus supporting its application in our larger cohort.

A key finding is a marked improvement in quality of life across various domains, as indicated by the WHOQOL-BREF and DLQI surveys. The results demonstrate not only statistically significant but also clinically relevant improvements, particularly in the physical and mental domains of the WHOQOL-BREF and in the DLQI total scores. These findings align with the hypothesis that BTA injections lead to substantial improvements in the management of facial hyperhidrosis, thus potentially increasing the quality of life [19].

The study notably places a strong emphasis on patient perception, offering a comprehensive view of the treatment’s utility. The improvements in patient-reported outcomes, such as the management of facial sweating and its impact on daily activities, are particularly insightful. This patient-centered approach underscores the importance of subjective experiences in evaluating treatment success, especially in conditions like facial hyperhidrosis, where physical symptoms are prominently visible and can lead to social and psychological distress [20,21,22].

The demographic profile of the study participants, predominantly younger individuals with a slightly higher representation of females and urban residents, offers valuable insights. This information is crucial for clinicians in tailoring treatment approaches for facial hyperhidrosis, considering the specific needs and contexts of different patient groups. Moreover, the correlation and regression analysis results reveal significant relationships between the severity of hyperhidrosis (HDSS), sweat production, and quality of life measures. The negative correlations between the DLQI total score and both HDSS and sweat production highlight the direct impact of symptom severity on the quality of life. Furthermore, the positive impact of younger age and improvements in the physical and mental domains of the WHOQOL on the DLQI total score offers a broader understanding of the factors influencing patient outcomes.

Similarly, a meta-analysis focused on gravimetric sweat rate reduction and quality-of-life outcomes, finding that BTA injections significantly reduced sweat production, with a 63% risk difference from baseline and improved disease severity and a 56% risk difference in the HDSS [23]. Quality-of-life, measured by the DLQI, also improved with a mean difference of 5.55. However, the analysis was limited to short-term outcomes over eight weeks, with a moderate overall risk bias, unlike our study, which covered a 12-month period. Similarly, a single-center study of five used the HDSS and the DLQI to assess the QOL in patients with hyperhidrosis and showed that BTA significantly reduced sweating for about 36 weeks, improving symptoms and life quality without severe side effects [24]. However, broader studies are needed to confirm these findings. Meanwhile, BTA remains a reliable treatment option for hyperhidrosis in various body areas, including fewer common regions and conditions like Frey syndrome and compensatory sweating [25].

Another study highlighted the complex interplay between the psychological aspects and the physical symptoms of hyperhidrosis, emphasizing that anxiety, stress, and social situations often exacerbate excessive sweating more than environmental factors like heat. This underscores hyperhidrosis as a year-round condition not limited to warmer seasons. Notably, the research found no significant differences in the psychosocial impacts of hyperhidrosis across different affected sites, such as clothing choices, social and emotional life, career, and hobbies [3,26]. Patients with hyperhidrosis at various sites faced similar burdens in managing their condition, regardless of the specific methods employed for symptom control or impact mitigation.

Other studies shed light on the daily challenges faced by individuals with hyperhidrosis, including extensive time spent managing symptoms and frequent clothing changes [27,28]. Moreover, one in five patients relied on accessories for daily life management. The findings suggest unmet healthcare needs among these individuals, such as access to treatment and support for the psychological aspects of the condition. This need for holistic care, including counseling, education, and psychotherapy, echoes challenges seen in other dermatologic conditions. The study utilized focus groups, semi-structured interviews, and online surveys to gather data, with each method contributing uniquely to the understanding of hyperhidrosis. While focus groups provided in-depth experiences, interviews offered more focused self-disclosure, and surveys brought a broader range of participant descriptions, together enhancing the validity of the research outcomes [29].

Although our study focused on all age groups, other research demonstrated that younger individuals and children are more likely to be psychologically affected by hyperhidrosis in their day-to-day lives [30,31]. Patients with hyperhidrosis often experience distress in social situations due to embarrassment and a sense of uncleanliness, leading to social anxiety, reduced self-effectiveness, and avoidance behaviors. This stress can exacerbate sweating, creating a vicious cycle. Such symptoms are particularly impactful in children, affecting their social and functional development with potential long-term effects, an area that warrants further exploration [32,33]. Hyperhidrosis not only complicates everyday tasks but also influences life choices, including hobbies, clothing, and occupation. While axillary hyperhidrosis is generally seen as more severe than palmar hyperhidrosis [34], some studies suggest that the overall impact of palmar, plantar, and axillary hyperhidrosis is similar [3]. This discrepancy highlights the need for more detailed studies to understand the quality of life differences based on symptom location, including less commonly discussed forms like craniofacial and plantar hyperhidrosis.

Hyperhidrosis is associated with an increased risk of cutaneous infections, atopic dermatitis, and other comorbidities such as migraine, asthma, obesity, and fatigue. The extent to which these comorbidities affect the quality of life in hyperhidrosis patients remains an area for further investigation. Early treatment of hyperhidrosis may prevent or mitigate these conditions, underscoring the importance of tailored treatments and monitoring for associated conditions in patients [27]. Despite the significant quality of life impact of hyperhidrosis, comparable to chronic diseases like psoriasis and eczema, it remains relatively understudied. Research comparing hyperhidrosis to other dermatological conditions reveals similar or even higher impacts on the emotional and functioning domains of patients’ lives. Yet, the severity and profound impact of hyperhidrosis on quality of life is not proportionately represented in the volume of research conducted.

The current study’s findings on the long-term utility of Botulinum toxin therapy in reducing symptom severity and sweat production, thereby improving the overall quality of life, are in line with the existing literature. This reinforces the notion that BTA injections can be a viable, less invasive alternative to surgical methods, offering both immediate relief and long-term benefits without significant risks or complications.

Nevertheless, our study exhibits certain limitations that warrant critical examination. First, the study’s patient cohort, drawn from specific clinical settings in Romania, may limit the generalizability of the findings. The demographic and geographic specificity of the participants might not fully represent the broader population suffering from facial hyperhidrosis, potentially affecting the applicability of the results in different contexts. Secondly, the exclusive focus on patients unresponsive to conventional therapies introduces a selection bias, as it excludes those who might have benefited from or preferred alternative treatments. This could skew the study’s outcomes towards more severe cases, potentially overestimating the treatment’s efficacy for the general population. Another limitation of the study is the potential methodological issue arising from the distance of six months between BTA injections, potentially causing an underestimation of the quality-of-life impact due to the resumption of symptoms when the effects of BTA wear off. This interval may not accurately capture the full fluctuation and duration of symptoms, leading to a possible underrepresentation of the true effects on quality of life. Additionally, the reliance on self-reported measures, while valuable for capturing patient perceptions, may be subject to subjective biases and variability in individual tolerance and reporting. Finally, the study’s observational design, though robust for longitudinal analysis, lacks the control of randomized trials, making it challenging to definitively attribute improvements solely to the treatment, as external factors could also influence the outcomes.

## 4. Conclusions

The study conclusively demonstrates that BTA therapy is a reliable long-term treatment option for facial hyperhidrosis, significantly improving patients’ quality of life. Over a one-year period, substantial reductions were observed in the HDSS scores, DLQI scores, and sweat production, with these improvements sustained at 12 months. Notably, the treatment positively impacted both physical symptoms and psychological well-being, as evidenced by improved WHOQOL-BREF scores in mental, physical, and social domains. The strong negative correlations identified between DLQI total scores, HDSS, and sweat production underscore the comprehensive utility of Botulinum toxin in addressing the multifaceted challenges posed by facial hyperhidrosis. These results endorse BTA as a viable option for managing the debilitating effects of this condition over the long term.

## 5. Materials and Methods

### 5.1. Research Design and Ethical Considerations

In this observational and longitudinal study, we aimed to investigate the effects of BTA treatment on the quality of life of facial hyperhidrosis patients across a year. Participants were enlisted from both private practices and the Dermatology Unit at Pius Brinzeu Clinical Emergency Hospital, associated with Victor Babes University of Medicine and Pharmacy, Timisoara, Romania. The research adhered to stringent ethical norms, receiving clearance from the Local Commission of Ethics for Scientific Research in line with EU GCP Directives 2005/28/EC, ICH standards, and the Declaration of Helsinki’s principles. Informed consent was obtained from all individuals, ensuring confidentiality and privacy throughout the study.

### 5.2. Inclusion and Exclusion Criteria

**Figure 4:**
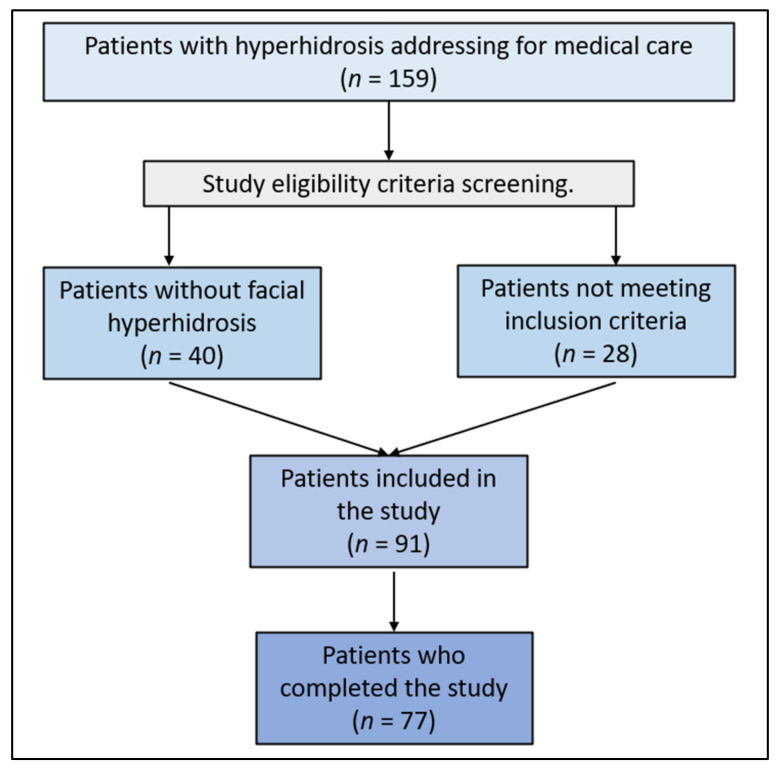
Study flowchart.

Individuals aged 18 and above, identified with primary facial hyperhidrosis that showed no response to other treatments, were selected for the study. Eligibility required a well-documented record of the condition spanning a minimum of six months before joining. Those excluded encompassed patients with secondary hyperhidrosis, a recent background of facial surgery, concurrent skin disorders, compromised immune systems, as well as pregnant or lactating individuals and those with a known allergy to Botulinum Toxin. Additionally, individuals who received any treatments for facial hyperhidrosis within the six months leading up to the start of the study were not considered. The participants who did not participate in all assessments and sessions according to the study protocol were excluded, as described in the study flowchart (Figure 4).

### 5.3. Variables and Procedures

The study spanned over a period of 12 months, with assessments conducted at baseline (before the first session and 1 month after the first session), at 6 months, and 1 year after the initial session. We allowed a 1-month duration between the BTA injections and the assessments to allow for an optimal effect of BTA treatment. Key variables included age, gender, duration of hyperhidrosis, sweat measurement, survey results, and treatment outcomes. BTA injections were administered by experienced dermatologists, with doses and techniques aligning with current best practices, where each patient received a 50 U IncoBTX-A dose per session with (Xeomin^®^, Merz Pharma, GmbH & Co kGaA, Frankfurt, Germany), diluted in 5 mL sterile 0.9% saline solution, with two sessions conducted (at study onset and 6 months).

The Injection technique was focused on targeting the deep dermis of the forehead or the junction with subcutaneous tissue, where sweat glands predominantly reside. BTA was injected intradermally to minimize muscle impact. Given the varied sweating patterns in facial hyperhidrosis, we customized the injection grid based on a gravimetric method using filter paper rather than using a standard approach with pre-defined injection sites. Post-injection, patients were monitored for immediate side effects and given follow-up care instructions.

Hyperhidrosis severity was evaluated using the Hyperhidrosis Disease Severity Scale (HDSS) [35]. Participants assessed their forehead perspiration on a scale where 1 represented “never noticeable”, 2 as “tolerable”, 3 as “barely tolerable”, and 4 as “intolerable”. A score of 3 or 4 was considered indicative of severe hyperhidrosis, whereas 1 or 2 indicated milder forms. Defining treatment efficacy involved decreasing the score from 4 or 3 to 2 or 1, denoting a considerable reduction in sweating. A relapse was identified by an increase of 1 point in the score after treatment. To quantitatively measure sweat secretion, the standardized filter paper was weighed before and after a 15-min application on the forehead, determining the rate of sweat in grams per 15 min. These assessments were conducted under controlled environmental conditions, maintaining room temperatures of 20–22 °C and humidity levels between 55–60%.

### 5.4. Surveys Employed

To gain a thorough insight into the experiences of the participants, various well-recognized instruments that were previously used and validated in the Romanian population and language were employed [36,37]. The 26-item WHOQOL-BREF questionnaire [36] was utilized to assess the overall quality of life. Additionally, the Dermatology Life Quality Index (DLQI) tool [38,39] was incorporated into this study to specifically gauge the effects of hyperhidrosis and to contrast these findings with more general quality of life evaluations. These instruments were administered online.

A non-standard survey was developed to delve deeper into the daily experiences of patients with facial hyperhidrosis, which comprised the following questions, with responses on a 1 to 10 scale:

1. Facial Sweating Severity: How severe is your facial sweating on a scale of 1 to 10, where 1 is no sweating, and 10 is extremely severe sweating?
2. Sweating Management: How well can you manage your facial hyperhidrosis-associated sweating? (1—not effectively at all, 10—extremely effectively)
3. Impact on Daily Activities: How much does facial hyperhidrosis interfere with your everyday activities? (1—no impact, 10—a significant impact)
4. Mood and Emotional Well-being: How do you rate your mood and emotional well-being overall? (1—very poor, 10—excellent)
5. Social Engagement: How comfortable are you in social situations while experiencing facial sweating? (1—very uncomfortable, 10—very comfortable)
6. Personal Relationships: How has facial sweating impacted your relationships with family, friends, etc.? (1—not affected at all, 10—significantly affected)
7. Professional and Educational Activities: How has facial hyperhidrosis affected your professional or educational pursuits? (1—no impact, 10—a significant impact)
8. Self-Confidence: What is your level of self-confidence in social and professional environments? (1—very low, 10—very high)
9. Sleep Quality: How has facial hyperhidrosis influenced the quality of your sleep? (1—very poor, 10—excellent)
10. Overall Quality of Life: How would you rate your overall life quality with facial hyperhidrosis? (1—very poor, 10—excellent).

The participants completed these surveys at the start of the study (baseline) and then again at specific intervals: 1 month post-treatment, 6 months after discharge, and at 12 months (following the second BTA injection). This systematic method ensured consistent monitoring and evaluation.

### 5.5. Software and Statistical Analysis

Data handling and analysis were carried out using SPSS version 26.0 (SPSS Inc., Chicago, IL, USA) statistical software. The number of participants was determined using a convenience sampling approach, targeting at least 56 individuals to achieve a 95% confidence level and a margin of error of 10%. Continuous variables were represented as mean ± standard deviation (SD), while categorical variables were expressed in terms of frequencies and percentages. To analyze the changes between more than two means of continuous variables, the ANOVA test was utilized. The Chi-square test was utilized for the categorical variables. Spearman’s correlation was used to determine associations between the study variables and quality of life factors, while a linear regression model was calculated to observe the most significant factors impacting the DLQI. A *p*-value threshold of less than 0.05 was set for statistical significance. All results were double-checked to ensure accuracy and reliability. The AI tool ChatGPT (OpenAI, San Francisco, CA, USA) was used for English grammar and spelling detection.

## Figures and Tables

**Table 1:** Patients’ background characteristics.

Variables	*n* = 77	%
Age (mean ± SD)	32.5 ± 9.8	-
Sex		
Male	35	45.5%
Female	42	54.5%
Area of residence		
Urban	47	61.0%
Rural	30	39.0%
Partnership status		
Divorced or Single	25	32.5%
In a relationship or Married	52	67.5%
Employment/educational status		
Working	50	64.9%
Studying	15	19.5%
None of the above	12	15.6%
Length of symptoms, months (mean ± SD)	18.2 ± 12.4	-
Severity of hyperhidrosis		
HDSS < 3	40	51.9%
HDSS ≥ 3	37	48.1%

SD—Standard Deviation; HDSS—Hyperhidrosis Disease Severity Scale (<3—less severe; ≥3—more severe).

**Table 2:** Longitudinal assessment of patient perception over quality of life.

Questions (1–10)	Before	At 1 Month	At 6 Months	At 12 Months	*p*-Value *
Facial Sweating Severity	7.5 ± 1.2	4.2 ± 0.9	5.0 ± 2.2	4.5 ± 1.1	<0.001
Sweating Management	5.1 ± 2.3	6.8 ± 1.4	7.5 ± 1.2	7.0 ± 3.5	<0.001
Impact on Daily Activities	6.4 ± 1.6	4.9 ± 2.1	5.1 ± 1.3	5.8 ± 1.9	<0.001
Mood and Emotional Well-being	6.5 ± 3.4	6.2 ± 2.3	6.7 ± 3.2	6.4 ± 3.1	0.779
Social Engagement	5.8 ± 2.5	6.3 ± 2.0	7.0 ± 3.1	7.1 ± 3.3	0.011
Personal Relationships	6.2 ± 1.6	6.8 ± 1.2	6.9 ± 2.4	6.5 ± 2.8	0.157
Professional and Educational Activities	5.5 ± 2.1	6.3 ± 2.8	6.6 ± 3.7	5.9 ± 3.5	0.138
Self-Confidence	5.9 ± 1.2	7.7 ± 1.3	7.9 ± 1.0	7.6 ± 1.2	<0.001
Sleep Quality	7.0 ± 1.8	7.1 ± 2.4	7.3 ± 2.1	6.8 ± 1.5	0.466
Overall Quality of Life	6.7 ± 3.5	7.4 ± 2.2	7.5 ± 1.3	7.2 ± 2.4	0.192

*—ANOVA test.

**Table 3:** Time-based evaluation of quality of life using WHOQOL-BREF questionnaire scores.

WHOQOL-BREF (Mean ± SD)	Before	At 1 Month	At 6 Months	At 12 Months	*p*-Value *
Physical domain	64.5 ± 5.2	65.3 ± 4.6	64.0 ± 4.8	66.5 ± 5.1	0.011
Mental domain	66.7 ± 6.1	70.2 ± 5.0	71.5 ± 6.7	70.8 ± 5.2	<0.001
Social domain	62.1 ± 5.7	65.9 ± 4.4	69.4 ± 4.9	68.7 ± 5.3	<0.001
Environmental domain	72.8 ± 6.3	72.0 ± 5.5	72.6 ± 5.2	71.9 ± 5.6	0.703

*—ANOVA test; SD—Standard Deviation; WHOQOL-BREF—Brief Version of the World Health Organization Quality of Life survey (higher scores indicate better quality of life).

**Table 4:** Longitudinal assessment of DLQI survey results in association with HDSS and sweat production.

Variables (Mean ± SD)	Before	At 1 Month	At 6 Months	At 12 Months	*p*-Value
DLQI total score	24.8 ± 4.2	6.2 ± 2.1	5.9 ± 2.3	6.5 ± 2.5	<0.001
HDSS	3.6 ± 0.5	1.2 ± 0.8	1.1 ± 0.7	1.3 ± 0.6	<0.001
Sweat production (g/15 min)	0.75 ± 0.15	0.18 ± 0.07	0.16 ± 0.05	0.20 ± 0.08	<0.001

ANOVA test; SD—Standard Deviation; DLQI—Dermatology Life Quality Index (higher scores indicate lower quality of life); HDSS—Hyperhidrosis Disease Severity Scale.

**Table 5:** Correlation matrix.

Variables (rho, *p*-Value)	DLQI Total Score	HDSS	Sweat Production (g/15 min)	WHOQOL-Physical	WHOQOL-Mental	WHOQOL-Social	WHOQOL-Environmental
DLQI Total Score	1						
HDSS	−0.72, <0.001	1					
Sweat Production (g/15 min)	−0.68, <0.001	0.65, <0.001	1				
WHOQOL-Physical	0.20, 0.194	−0.31, 0.016	−0.19, 0.430	1			
WHOQOL-Mental	0.55, <0.001	−0.49, 0.002	−0.54, 0.002	0.68, <0.001	1		
WHOQOL-Social	0.33, 0.062	−0.61, <0.001	−0.56, <0.001	0.51, 0.001	0.49, <0.001	1	
WHOQOL-Environmental	0.18, 0.355	−0.26, 0.193	−0.13, 0.386	0.35, 0.092	0.52, <0.001	0.47, <0.001	1

Spearman’s rho is used due to the ordinal nature of some variables.

**Table 6:** Regression analysis for DLQI total score.

Variables	B Coefficient	Standard Error	Beta	t-Value	*p*-Value
Constant	15.2	2.1	-	7.24	<0.001
HDSS	−3.8	0.6	−0.45	−6.33	<0.001
Sweat Production (g/15 min)	−2.2	0.5	−0.3	−4.4	<0.001
Age	−0.1	0.03	−0.18	−3.33	0.001
Sex (1 = Male, 0 = Female)	1.2	0.8	0.09	1.5	0.136
Urban Residence (1 = Urban, 0 = Rural)	−0.6	0.7	−0.05	−0.85	0.396
WHOQOL-Physical	0.3	0.1	0.15	3.0	0.003
WHOQOL-Mental	0.2	0.1	0.12	2.0	0.046

Dependent Variable—DLQI Total Score; B: Unstandardized regression coefficient; Beta: Standardized regression coefficient.

## Data Availability

Data available on request.
